# Secondary Lingular Sleeve Resection to Avoid Pneumonectomy Following Bronchial Anastomotic Dehiscence after Left Lower Lobe Sleeve Resection for Destroyed Lung Syndrome

**DOI:** 10.1055/s-0038-1635124

**Published:** 2018-02-27

**Authors:** Servet Bölükbas, Robert Zanner, Michael Eberlein, Christian Biancosino, Bassam Redwan

**Affiliations:** 1 Department of Thoracic Surgery, Kliniken Essen-Mitte, Evang, Huyssens-Stifftung/Knappschafts-Krankenhaus, Essen, Germany; 2 Department of Anaesthesiology, Klinikum rechts der Isar, Technische Universität München, Munich, Germany; 3 Division of Pulmonary, Critical Care and Occupational Medicine, University of Iowa Hospitals and Clinics, Iowa City; 4 Department of Thoracic Surgery, Helios University Hospital Wuppertal, Wuppertal, Germany; 5 Division of Thoracic Surgery, and Lung Transplantation, University Hospital Münster, Münster, Germany

**Keywords:** sleeve resection, anastomotic dehiscence, complication

## Abstract

Bronchial sleeve resections are technically demanding procedures compared with lobectomies. In case of bronchial anastomotic dehiscence, secondary pneumonectomy is the treatment of choice. However, a secondary pneumonectomy is usually associated with high morbidity and mortality. Here, we first report, to the best of our knowledge, a secondary lingular sleeve resection following bronchial anastomotic dehiscence after left lower lobe sleeve resection in a patient with a destroyed lobe syndrome due to a pseudotumor. This approach enabled the avoidance of secondary pneumonectomy, hence reducing the possible pneumonectomy-associated complications.

Bronchial sleeve resections are performed to avoid pneumonectomy in centrally located lung tumors. These procedures are technically demanding compared with lobectomies. Several studies have demonstrated an acceptable long-term survival following bronchial sleeve resections with low mortality and bronchial anastomotic complication rates. 1 2 Here we first describe a technique of secondary lingular sleeve resection to avoid pneumonectomy after bronchial anastomosis dehiscence following a sleeve resection of the left lower lobe (LLL).

## Case Presentation and Surgical Technique

**Fig. 1:**
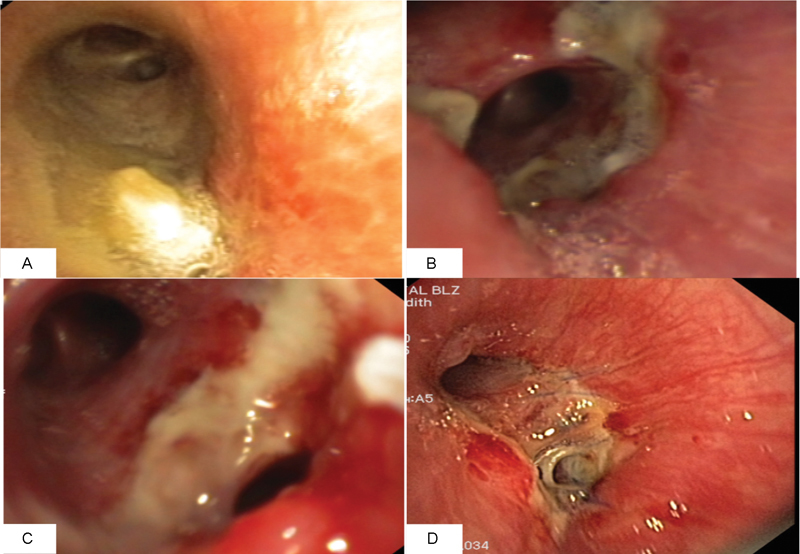
Flexible bronchoscopy showing the exophytic tumor occluding the left lower bronchus ( **A** ). Bronchial anastomosis dehiscence after left lower sleeve resection on postoperative day 10 ( **B** – **C** ). Endobronchial finding prior to discharge after secondary lingular sleeve resection ( **D** ).

**Fig. 2:**
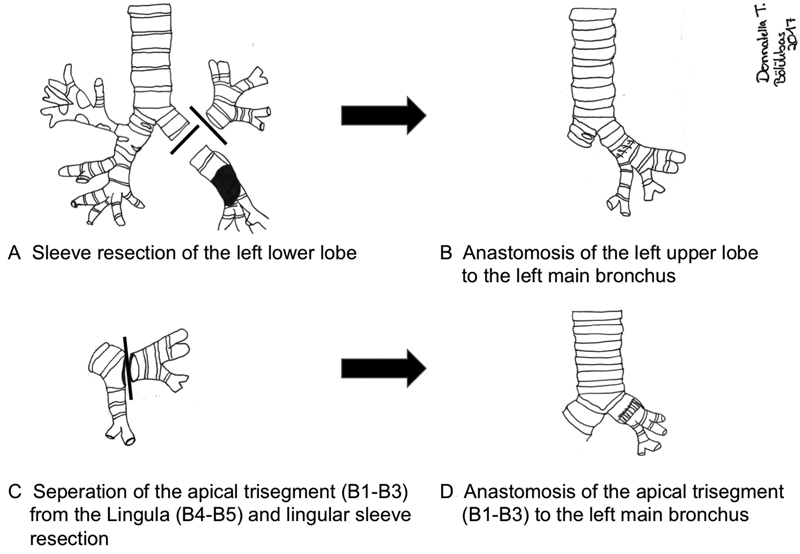
Schematic figure of the left lower sleeve resection ( **A** , **B** ) and subsequent lingular sleeve resection ( **C** , **D** ).

**Fig. 3:**
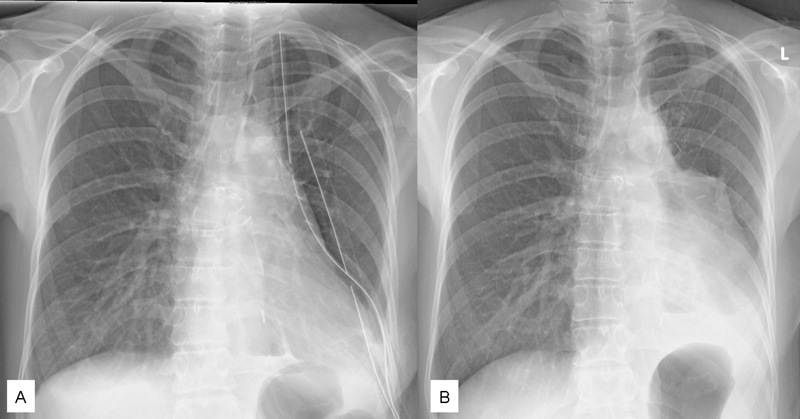
Postoperative X-ray of the chest immediately after sleeve resection of the left lower lobe ( **A** ) and before discharge after the redo-sleeve resection ( **B** ).

A 36-year-old female patient was admitted to our hospital for recurrent pulmonary infections. Flexible bronchoscopy revealed an exophytic tumor completely occluding the left lower lobe bronchus (LLLB) ( Fig. 1A ). Biopsy was performed. However, histological examination was highly suspicious but not confirmatory for malignancy. Computed tomography (CT) scan of the chest showed a total atelectasis of the LLL with the radiological signs of a destroyed lobe syndrome. A positron emission tomography CT scan revealed purulent fusion of the LLL. Increased fludeoxyglucose uptake was not observed otherwise. Despite antibiotic therapy, no improvement occurred. Therefore, surgical resection was considered. Due to the exophytic tumor in the LLB, full circumferential bronchial resection of the LLL was performed in concordance with a standardized surgical approach. 3 Special care was taken to preserve the nutritional layer of the bronchi. Routinely, full hilar release including semicircular pericardiotomy was performed to facilitate tension-free anastomosis. Bronchial anastomosis between the left upper lobe and left main bronchus (LMB) was achieved by interrupted 4–0 polydioxanone sutures (PDS, Ethicon, Germany) tied at completion of the anastomosis ( Fig. 2A , B ). The knots were placed outside the lumen. The patient was extubated at the operation room and transferred to the intensive care unit. The postoperative X-ray showed a fully expanded left upper lobe ( Fig. 3A ).

Final pathohistological examination revealed destroyed lung syndrome due to endobronchial pseudotumor. We routinely perform two postoperative bronchoscopies to monitor the bronchial healing. 3 The first bronchoscopy on postoperative day (POD) 7 showed an intact bronchial anastomosis with slight fibrin deposits. The healing was classified as grade 2 according to the classification of tracheobronchial anastomoses. 4 On POD 10, bronchial anastomotic dehiscence at the medial wall of the anastomosis was detected during second routine bronchoscopy ( Fig. 1B , C ). Therefore, surgical revision was indicated. Intraoperatively, the ischemic and inflamed bronchial edges from the LMB and left upper bronchus (LUB) were resected as described elsewhere. 5 However, the distal LUB was still edematous and inflamed. Under these circumstances, reanastomosis of the LUB with the LMB was not favored. To avoid pneumonectomy, anatomical lingular resection with subsequent telescope anastomosis of the apical trisegment group (B1–3) with the LMB was performed. Bronchial anastomosis was achieved as described above ( Fig. 2C , D ). The postoperative course was uneventful. Flexible bronchoscopy revealed healing of the anastomosis ( Fig. 1D ) and the patient was discharged uneventfully ( Fig. 3B ).

## Comment

Bronchial anastomotic dehiscence represents one of the major and often lethal complications after bronchial sleeve resection. In a meta-analysis, mortality and morbidity after sleeve lobectomy varied between 0% to 10.5% and 11.1% to 59.7%, respectively. 6 Bronchial anastomotic dehiscence might be found in 5% to 8.7% after sleeve lobectomy. 7 The risk might be enhanced especially in inflammatory conditions or after induction treatment for nonsmall cell lung cancer. However, if the surgeon is concerned about the bronchial healing, vascularized tissue might be used to wrap and protect the anastomosis, for example, pedicle flap of the omentum, intercostal muscle flaps, pleural flaps, or pericardial flaps. 8

Secondary completion pneumonectomy is generally the treatment of choice for anastomotic dehiscence. However, completion pneumonectomy is usually associated with high operative mortality of up to 67%. 1 2 8 Furthermore, completion pneumonectomy might be contraindicated due to loss of pulmonary reserves and functional reasons.

To the best of our knowledge, this is the first report of secondary lingular sleeve resection following bronchial anastomotic dehiscence after LLL sleeve resection. We sacrificed the lingula to rescue the apical trisegment group (B1–3). This extended redo-sleeve resection enabled the avoidance of pneumonectomy, hence reducing the possible pneumonectomy-associated complications as well as saving pulmonary reserves and quality of life. In experienced hands, redo-sleeve resections might be an option for the management of postoperative bronchial anastomotic dehiscence.
